# Automated Pathologic TN Classification Prediction and Rationale Generation From Lung Cancer Surgical Pathology Reports Using a Large Language Model Fine-Tuned With Chain-of-Thought: Algorithm Development and Validation Study

**DOI:** 10.2196/67056

**Published:** 2024-12-20

**Authors:** Sanghwan Kim, Sowon Jang, Borham Kim, Leonard Sunwoo, Seok Kim, Jin-Haeng Chung, Sejin Nam, Hyeongmin Cho, Donghyoung Lee, Keehyuck Lee, Sooyoung Yoo

**Affiliations:** 1 ezCaretech Research & Development Center Seoul Republic of Korea; 2 Department of Radiology Seoul National University Bundang Hospital Seongnam Republic of Korea; 3 Office of eHealth Research and Business Seoul National University Bundang Hospital Seongnam Republic of Korea; 4 Department of Pathology Seoul National University College of Medicine Seoul Republic of Korea; 5 Department of Pathology and Translational Medicine Seoul National University Bundang Hospital Seongnam Republic of Korea; 6 Department of Family Medicine Seoul National University Bundang Hospital Seongnam Republic of Korea

**Keywords:** AJCC Cancer Staging Manual 8th edition, American Joint Committee on Cancer, large language model, chain-of-thought, rationale, lung cancer, report analysis, AI, surgery, pathology reports, tertiary hospital, generative language models, efficiency, accuracy, automated

## Abstract

**Background:**

Traditional rule-based natural language processing approaches in electronic health record systems are effective but are often time-consuming and prone to errors when handling unstructured data. This is primarily due to the substantial manual effort required to parse and extract information from diverse types of documentation. Recent advancements in large language model (LLM) technology have made it possible to automatically interpret medical context and support pathologic staging. However, existing LLMs encounter challenges in rapidly adapting to specialized guideline updates. In this study, we fine-tuned an LLM specifically for lung cancer pathologic staging, enabling it to incorporate the latest guidelines for pathologic TN classification.

**Objective:**

This study aims to evaluate the performance of fine-tuned generative language models in automatically inferring pathologic TN classifications and extracting their rationale from lung cancer surgical pathology reports. By addressing the inefficiencies and extensive parsing efforts associated with rule-based methods, this approach seeks to enable rapid and accurate reclassification aligned with the latest cancer staging guidelines.

**Methods:**

We conducted a comparative performance evaluation of 6 open-source LLMs for automated TN classification and rationale generation, using 3216 deidentified lung cancer surgical pathology reports based on the *American Joint Committee on Cancer (AJCC) Cancer Staging Manual* *8th edition*, collected from a tertiary hospital. The dataset was preprocessed by segmenting each report according to lesion location and morphological diagnosis. Performance was assessed using exact match ratio (EMR) and semantic match ratio (SMR) as evaluation metrics, which measure classification accuracy and the contextual alignment of the generated rationales, respectively.

**Results:**

Among the 6 models, the Orca2_13b model achieved the highest performance with an EMR of 0.934 and an SMR of 0.864. The Orca2_7b model also demonstrated strong performance, recording an EMR of 0.914 and an SMR of 0.854. In contrast, the Llama2_7b model achieved an EMR of 0.864 and an SMR of 0.771, while the Llama2_13b model showed an EMR of 0.762 and an SMR of 0.690. The Mistral_7b and Llama3_8b models, on the other hand, showed lower performance, with EMRs of 0.572 and 0.489, and SMRs of 0.377 and 0.456, respectively. Overall, the Orca2 models consistently outperformed the others in both TN stage classification and rationale generation.

**Conclusions:**

The generative language model approach presented in this study has the potential to enhance and automate TN classification in complex cancer staging, supporting both clinical practice and oncology data curation. With additional fine-tuning based on cancer-specific guidelines, this approach can be effectively adapted to other cancer types.

## Introduction

Pathologic staging is a crucial part of cancer management because it provides vital information about the extent of the disease through histopathological examination [1]. Lung cancer continues to be the leading cause of cancer-related mortality globally, surpassing the combined deaths from colon, breast, and prostate cancers, underscoring its poor prognosis [2]. Therefore, pathologic staging is particularly important for tailoring treatment strategies and accurately predicting patient outcomes [3,4]. Accurate staging is essential in high-mortality cancers like lung cancer, as it enables clinicians to select the most appropriate and effective treatment strategies based on the tumor’s specific characteristics and extent of spread. This precision ensures that treatment is tailored to the cancer’s stage, significantly influencing survival outcomes and improving prognosis. It is typically performed manually in accordance with the *American Joint Committee on Cancer (AJCC) Cancer Staging Manual* specific for each cancer type. This manual process is prone to human error and requires considerable time and effort to ensure accuracy and compliance with the latest AJCC guidelines. Data-entry errors can result in inaccurate patient records, potentially affecting treatment decisions and research outcomes. Manual staging has the drawback of potential inconsistencies in interpretation among pathologists, which can impact reliability. Leveraging automated staging methods can improve consistency and reliability, particularly in large-scale studies. Additionally, the AJCC staging guidelines have undergone multiple updates since the release of the 6th edition in 2003, including the 7th edition in 2010, the 8th edition in 2018, and the forthcoming 9th edition. Each update introduces the potential for inconsistencies when integrating data from previous versions. Therefore, updating or verifying the staging data to align with the current AJCC guidelines is labor-intensive and can cause delays in clinical decision-making.

These challenges necessitate the development of automated technologies that can directly classify pathologic stages from textual pathology reports. These technologies would streamline the staging process, reduce human errors, and ensure consistent application of the latest guidelines. Thus, automated data extraction systems can enhance the efficiency and reliability of clinical and research processes.

The automation of this process has primarily relied on rule-based natural language processing (NLP) techniques [5,6]. However, these approaches have inherent limitations, as they require the manual creation of rules to extract information, followed by an additional step to map the extracted data to TN classification criteria. This process is prone to errors and inefficiencies, and slight variations in the context or expressions used in pathology reports can hinder these methods from effectively handling complex linguistic features. To address these challenges, advanced techniques, such as large language models (LLMs), hold the potential to automatically comprehend context, infer pathologic staging, and provide transparent rationales.

Recent studies have actively explored the application of language models in medical and clinical information extraction [7-11]. In particular, research has been conducted using Bidirectional Encoder Representations from Transformers (BERT) [11,12], a language model based on the transformer architecture [13]. For example, Hu et al [11] developed a system to extract information from lung cancer computed tomography reports according to the 8th edition TNM classification. To establish lung cancer staging, they selected 14 key entities, including tumor shape, density, and invasion, embedded the computed tomography reports using word2vec [14], and performed named entity recognition (NER) for each entity using a combination of BERT and bidirectional long short-term memory. In the study, NER with BERT demonstrated excellent performance in information extraction, achieving a macro *F*_1_-score of 0.901 and a micro *F*_1_-score of 0.946. Zhou et al [12] also fine-tuned Blue-BERT [15] with cancer-specific terminology to develop CancerBERT, defining eight key phenotypes, such as tumor size, cancer grade, histological type, and cancer stage, to support clinical decision-making in patients with breast cancer. CancerBERT was trained to automatically recognize these phenotypes in clinical and pathology reports, achieving a micro–*F*_1_-score of 0.909 in NER performance for these eight phenotypes, demonstrating superior performance.

While deep learning–based methods show effective results in information extraction using NER, they still have limitations in extracting implicit information embedded in sentences. Additionally, NER-based approaches often require further postprocessing, such as relationship classification between extracted entities, and are less robust to typos and synonyms, making dictionary creation time-consuming and costly.

Recent advances in LLMs have demonstrated strengths in understanding various contexts and handling diverse expressions, making them suitable for information extraction [16]. In particular, prompt engineering enables LLMs to generate more accurate outputs when specific outputs are needed [17], and zero-shot prompting allows the model to flexibly solve new problems, making it advantageous for application across different domains [18]. However, studies leveraging LLMs to provide TN classification and rationales based on specific cancer staging guidelines remain limited.

Advances in prompt engineering have paved the way for the chain-of-thought (CoT) prompting approach [19]. CoT prompts enable complex reasoning by incorporating intermediate reasoning steps. Furthermore, CoT fine-tuning can improve the zero-shot learning performance of language models [20-22]. Models that have been fine-tuned using the CoT method can provide their “rationale” by describing the intermediate reasoning steps involved in solving a problem. This not only demonstrates the results but also aids in understanding and interpreting the decisions of the model. Providing the “rationale” is particularly beneficial in fields where evidence-based decisions are crucial, such as health care, because it facilitates the review and acceptance of the decisions of the model by health care professionals. TN classification in pathologic staging requires the integration of various clinical factors and a clearly defined logical process. However, traditional rule-based NLP approaches are limited by their inability to explicitly perform complex reasoning, relying instead solely on predefined rules for information extraction. CoT prompting addresses this limitation by enabling the model to explicitly present intermediate reasoning steps and logical justifications during the problem-solving process. This approach enhances both the consistency and interpretability of the pathologic TN classification task.

This study proposes a method for automatically inferring TN classification and its rationales based on the *AJCC Cancer Staging Manual 8th edition* [23] using a generative language model (GLM) and evaluates its performance in lung cancer surgical pathology reports. We focused on demonstrating the applicability of lightweight GLMs for addressing inferential tasks, such as predicting pathologic TN classification, in environments with limited computing resources, such as medical institutions. Lightweight models, with their lower memory and processing requirements, provide faster inference speeds and minimize the need for expensive hardware upgrades. These characteristics make them well suited for real-time clinical use and deployment across diverse medical institutions with varying levels of technical infrastructure.

This study aims to develop a generative LLM-based approach for automated pathologic TN classification that also provides interpretable rationales, potentially transforming pathology report analysis in lung cancer care.

## Methods

### Data Description

This is a retrospective observational study using electronic health records (EHRs). The retrospective EHR data were selected for their extensive dataset availability and their capacity to provide detailed clinical information, facilitating the evaluation and validation of LLMs for TN classification and rationale extraction. We used EHRs obtained from Seoul National University Bundang Hospital (SNUBH) between May 2003 and December 2021 in this study. These records were extracted from the Observational Medical Outcomes Partnership Common Data Model data [24,25]. The Observational Medical Outcomes Partnership Common Data Model database contains comprehensive data, including basic patient information, health care records, family history, diagnoses, drug exposure, test results, biomarkers, surgeries, and procedures. All reports were stored in text format within the NOTE table, from which we selected deidentified 7832 surgical pathology reports from patients with lung cancer (*International Classification of Diseases, Tenth Revision* diagnosis code: C34). To ensure high reliability in data extraction from lung cancer surgical pathology reports, validation was obtained from a pathologist at SNUBH rather than relying on external data. As SNUBH data can be verified by internal domain experts and originates from a tertiary general hospital that adheres to international guidelines and standards, this data source was chosen to ensure high-quality data.

### Ethical Considerations

This study was approved as exempt by the institutional review board of Seoul National University Bundang Hospital because of the use of deidentified patient data in a secure environment (X-2404-897-902).

### Data Preprocessing

**Figure 1:**
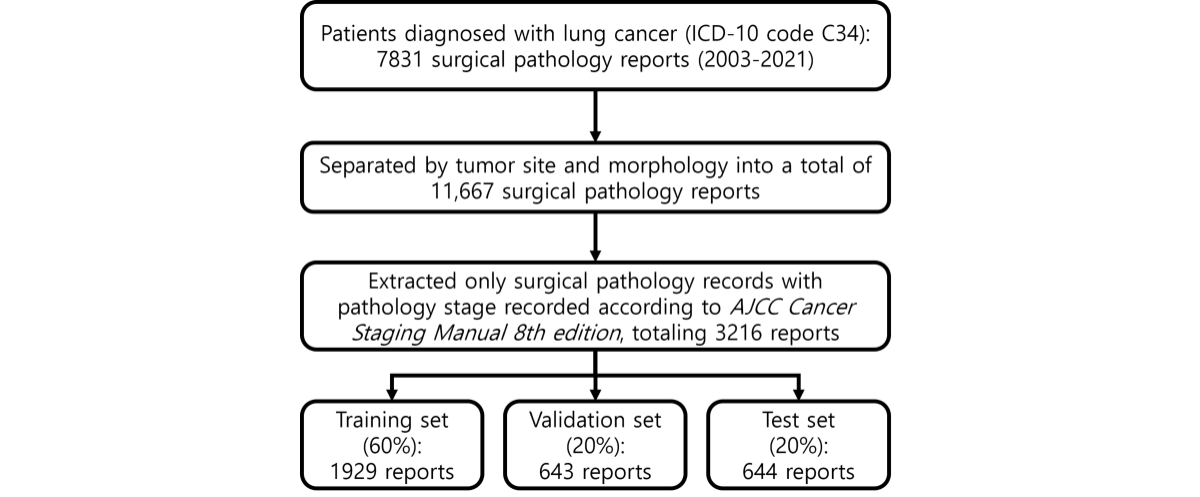
Data preprocessing flow. AJCC: American Joint Committee on Cancer; ICD-10: International Classification of Diseases, Tenth Revision.

Figure 1 shows the flowchart of the data preprocessing. A single surgical pathology report of lung cancer typically contains at least one lesion. In this study, a dataset of lung cancer surgical pathology reports was constructed using the following separation process.

First, we selected 7831 surgical pathology reports from patients who had undergone surgery and were diagnosed with lung cancer, as indicated by the *International Classification of Diseases, Tenth Revision* code C34. Second, since the lung cancer surgical pathology reports contained information on multiple lesion locations, we separated the report for each lesion individually. For example, a report containing two parts—“[A] Lung, right upper lobe, lobectomy” and “[B] Lung, left lower lobe, lobectomy”—was separated into individual lesions. Third, the reports, now separated by lesion location, were further divided based on the diagnosis of morphology, resulting in a total of 11,667 reports. For example, the “[B] Lung, left lower lobe, lobectomy” part included subparts such as “1. ADENOCARCINOMA, acinar predominant” and “2. Atypical alveolar pneumocytic hyperplasia.” These subparts were divided according to the diagnosis of morphology. The pathology reports used in this study did not include patient names or identification numbers, and identifiers such as pathology numbers were removed to ensure the data was deidentified. Consequently, personal information, such as patient names, was also not included in the sections containing lesion information.

Reports compiled prior to 2017 were excluded from the final dataset, as they were based on the *AJCC Cancer Staging Manual 6th and 7th edition* criteria, which do not align with the *AJCC Cancer Staging Manual 8th edition* criteria used in this study. Due to differences in TN classification criteria across *AJCC Cancer Staging Manual* editions, the same malignant tumor may be assigned different TN classifications depending on the version, potentially leading to discrepancies. We then constructed a final dataset from 3216 reports compiled in accordance with the *AJCC Cancer Staging Manual 8th edition*, an internationally recognized guideline for systematic cancer staging that undergoes regular reviews and revisions [26]. This manual is used to classify the extent of cancer progression based on factors such as tumor size, location, invasion of adjacent tissues, lymph node metastasis, and distant metastasis.

### Label Assignment

#### Overview

**Figure 2:**
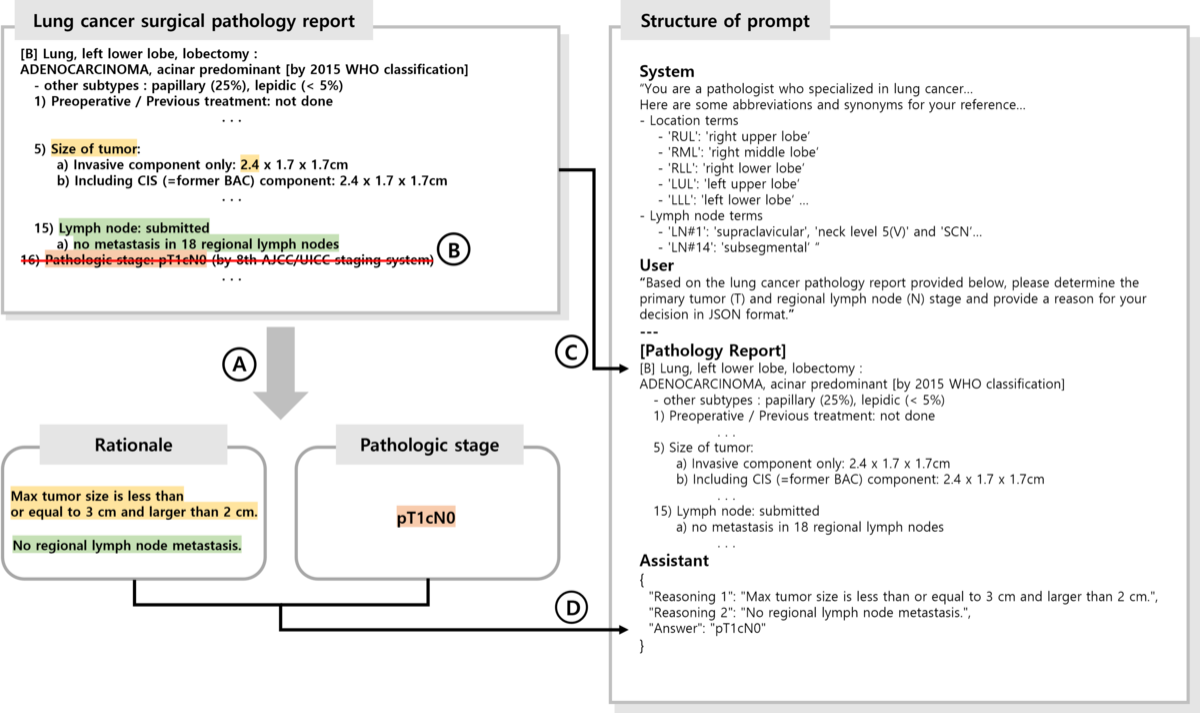
Label assignment and structure of prompt. (A) Identified and labeled both the recorded pathologic TN stage and the rationale sentences determining TN classification, (B) Removed sentences containing the recorded TN stage, (C) Included the preprocessed pathology report in the prompt, (D) Included the labeled elements from (A) in the prompt.

Figure 2 shows the labeling process, which is divided into two parts. First, we labeled rationale sentences containing evidence for TN staging. We then identified the TN staging results from the lung cancer surgical pathology reports and labeled the TN classifications accordingly.

#### Rationale Labeling

We identified rationale sentences in the lung cancer surgical pathology reports that determine the TN classification (Multimedia Appendix 1), and these sentences were classified according to the *AJCC Cancer Staging Manual 8th edition* criteria. The identified rationale sentences were then used as labels for model training. This process was crucial for generating sentences that reflected a deep understanding of the TN classification decision and were included in the dataset.

#### Pathologic TN Classification Labeling

We identified sentences from lung cancer surgical pathology reports in which the TN pathologic stage was recorded. The pathologic stage was determined based on the identified data. Next, we removed sentences in which the TN stage was recorded from the reports to prevent that information from being used to train the model. For example, as shown in Figure 2, we identified the TN stage “pT1cN0” from a sentence in a lung cancer surgical pathology report, constructed the label, and deleted that sentence.

Two health care professionals constructed a gold standard for the labeling process through an in-depth review and validation based on their clinical experience.

### Train Data

Figure 2 shows the prompt for fine-tuning using the CoT approach. The initial section of the prompt structure provides an explanation of the task, along with a list of abbreviations and synonyms related to lung lobes and lymph nodes. The middle section contains the lung cancer surgical pathology report, whereas the final section contains the pathologic stage and rationale data obtained through labeling. The LLM used these structured prompts to identify crucial information from the lung cancer surgical pathology reports and perform the necessary logical reasoning for generating TN classification. The model was trained to use the JSON structure in its output format during fine-tuning. This enabled the automatic evaluation of experimental results, as it standardized the varied forms of the output generated by the model.

The dataset used in this study consisted of 3216 reports, with 1929 allocated to the training set, 643 to the validation set, and 644 to the test set. Stratified random sampling was used to ensure a balanced representation across TN classifications. Reports were stratified by specific TN classifications, and each subset (training, validation, and test) was constructed to reflect the proportional distribution of these classifications. This approach aimed to include diverse cases from all TN categories, enabling the model to generalize effectively across various classifications.

### Model Selection

The primary objective of this study was to evaluate the applicability and performance of relatively lightweight GLMs in environments with limited computing resources, such as medical institutions. To achieve this, we selected two representative open-source GLMs, Llama and Mistral, which are freely available and facilitate flexible deployment in clinical settings without licensing constraints. Additionally, the Orca2 model, a fine-tuned version of Llama-2, was included. Orca2 is enhanced with robust reasoning capabilities, developed using a synthetic dataset filtered and fine-tuned from the FLAN-v2 Collection [27]. Optimized with various reasoning techniques, Orca2 demonstrates excellent performance in generating step-by-step responses to complex questions. Models with parameter sizes ranging from 7B to 13B were chosen to examine lightweight options suitable for resource-constrained clinical environments and the demands of complex cancer staging. The selected models were Orca2_ 7 B [22], Orca2_ 13 B [22], Mistral_ 7 B [28], Llama2_ 7 B [29], Llama2_ 13 B [29], and Llama3_8b.

### Model Fine-Tuning

The training process comprised 6000 steps, with each graphics processing unit processing a batch size of 2. Gradient accumulation was performed every four steps, and the learning rate was set to 1.5e-5. We implemented a low-rank adaptation to use the fine-tuning technique [30]. Low-rank adaptation is a part of parameter-efficient fine-tuning [31] that preserves the overall model structure and learned patterns while substantially reducing the number of parameters required for training in specific areas of the model. Based on initial testing results, we selected a configuration with γ and α values set to 32, effectively balancing training efficiency and model performance. Additionally, through preliminary experiments evaluating various configurations, we determined that a dropout rate of 0.05 was optimal for maintaining model stability while preventing overfitting. We used the cross-entropy loss function and AdamW 32-bit optimizer during training. The model with the lowest validation loss was selected. We minimized randomness by setting the top k to 1 and restricting the beam search to 1 during the evaluation phase. For a summary of the experimental setup for LLM training, see Multimedia Appendix 2.

### Evaluation

#### Overview

In this study, we evaluated the performance of a fine-tuned GLM in the automatic prediction of TN classifications and their rationale sentences for lung cancer surgical pathology reports. We used precision, recall, and *F*_1_-score, focusing on the accuracy of the sentences produced to assess the performance of the GLMs. Additionally, we introduced the exact match ratio (EMR) and semantic match ratio (SMR) as evaluation metrics. These metrics were chosen because they are more appropriate for clinical text generation than conventional NLP metrics like bilingual evaluation understudy or recall-oriented understudy for gisting evaluation. In clinical contexts, even small differences in terms, numbers, or classifications can significantly impact the medical interpretation of a sentence. Since metrics like bilingual evaluation understudy and recall-oriented understudy for gisting evaluation measure surface-level similarity, they could give high scores even if critical clinical information, such as tumor size or stage, differs. Therefore, EMR and SMR were selected to assess whether the generated sentences precisely match the *AJCC Cancer Staging Manual 8th edition* criteria and maintain consistent clinical meaning. To this end, we used the following evaluation metrics:

#### EMR

EMR represents the ratio of sentences generated by a model that perfectly matches the standard answer. This was defined as follows:

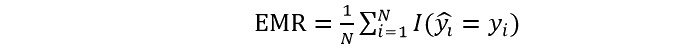



where *N* represents the total number of samples, *I*(°) is an indicator function that equals 1 if the condition inside the parentheses is true and 0 if false, 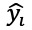. represents the model’s prediction, and *y_i_* represents the actual value.

#### SMR

The SMR represents the ratio of sentences generated by the model that have the same contextual meaning as the standard answer, even if they are not a perfect match. For example, if the standard answer is “Regional lymph node metastasis information is missing or not submitted” and the generated rationale sentence is “Regional lymph node metastasis information is missing or not insufficient,” an expert would validate whether it matches in context. SMR is defined as follows:

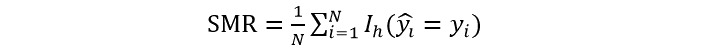



where *I_h_*(°) represents expert validation, which assesses whether the generated sentence and standard answer match in context. The validation process was carried out by two domain experts. They assessed whether the generated rationale sentences contextually matched the TN classification criteria according to the *AJCC Cancer Staging Manual 8th edition* for determining the stage. To ensure consistency, the experts followed a standardized validation protocol based on the AJCC guidelines and verified the contextual alignment of each rationale sentence with the corresponding TN classification.

We excluded cases that did not fit the definition of the patient cohort as confirmed patients with lung cancer, such as those with T0 (no tumor) and Tx (uncertain tumor presence). The T classifications included Tis, T1mi, T1a, T1b, T1c, T2a, T2b, T3, and T4 whereas N classifications included Nx, N0, N1, and N2. The National Comprehensive Cancer Network (NCCN) guidelines (version 5.2024) for nonsmall cell lung cancer [32] recommend definitive concurrent chemoradiation as the standard treatment for N3 disease, not upfront surgery. Because N3 disease is not an indication for surgery, it was not observed in the lung cancer surgical pathology reports.

Cases that did not align with the TN classification criteria of the *AJCC Cancer Staging Manual 8th edition* were identified as out-of-scope (OOS) and excluded from the study. Specifically, cases corresponding to TN categories from the 6th or 7th editions were classified as OOS. For instance, in the *AJCC Cancer Staging Manual 8th edition*, the T2 category is subdivided into T2a and T2b, whereas such subdivisions were absent in earlier editions. During the evaluation process, if the model provided only the answer “T2” without further subdivision, it was deemed inconsistent with the study’s objectives, which adhere to the 8th edition criteria, and was thus classified as OOS. In addition to the subclassification issue of T2, the TN classification criteria in previous editions differ from those of the *AJCC Cancer Staging Manual 8th edition*. For example, a 5 cm lung cancer that has not invaded surrounding tissues is classified as “T2a” in the *AJCC Cancer Staging Manual 7th edition* but as “T3” in the 8th edition, potentially leading to misinterpretation of test results. Furthermore, cases in which the model failed to provide any TN classification answer were also categorized as OOS. This OOS identification method was verified by cross-referencing the *AJCC Cancer Staging Manual 8th edition* guidelines and classification results, ensuring that only relevant cases were included in the analysis. The number of OOS cases and the OOS ratio for the test set were recorded, providing a quantitative measure of the model’s ability to adhere to the current classification criteria.

## Results

### Overview

We evaluated the performance of different GLMs fine-tuned to infer the TN classification and rationale sentences in this study. Six models (Orca2_7b, Orca2_13b, Mistral_7b, Llama2_7b, Llama2_13b, and Llama3_8b) were evaluated. We partitioned the dataset into training, validation, and test sets to assess the performance of each model.

### Dataset Distribution

The distribution of data per set is presented in Table 1. Model training and evaluation were conducted based on this distribution.

**Table 1:** Distribution of TN categories across training, validation, and test sets, showing the count and percentage of each category in each dataset.

TN categories	Training set (n=1929), n (%)	Validation set (n=643), n (%)	Test set (n=644), n (%)
Tis	20 (1.04)	6 (0.93)	12 (1.86)
T1mi	175 (9.07)	64 (9.95)	51 (7.92)
T1a	219 (11.35)	66 (10.26)	76 (11.80)
T1b	476 (24.68)	154 (23.95)	154 (23.91)
T1c	257 (13.32)	94 (14.62)	94 (14.60)
T2a	468 (24.26)	145 (22.55)	149 (23.14)
T2b	91 (4.72)	36 (5.60)	34 (5.28)
T3	165 (8.55)	63 (9.80)	66 (10.25)
T4	58 (3.01)	15 (2.33)	8 (1.24)
Nx	210 (10.89)	80 (12.44)	81 (12.58)
N0	1389 (72.01)	436 (67.81)	452 (70.19)
N1	154 (7.98)	64 (9.95)	50 (7.76)
N2	176 (9.12)	63 (9.80)	61 (9.47)

### Performance on Pathologic TN Classification

Table 2 presents the EMR for the TN classification prediction of each model. To evaluate TN classification, we considered cases in which both the T and N classifications were accurate, as well as those in which either the T or N classification was accurate. The Orca2_13b model demonstrated the highest performance, with EMRs of 0.934, 0.936, and 0.998 for TN, T, and N classifications, respectively. The Orca2_7b model also exhibited competitive performance, with EMRs of 0.914, 0.917, and 0.996 for TN, T, and N classifications, respectively.

**Figure 3:**
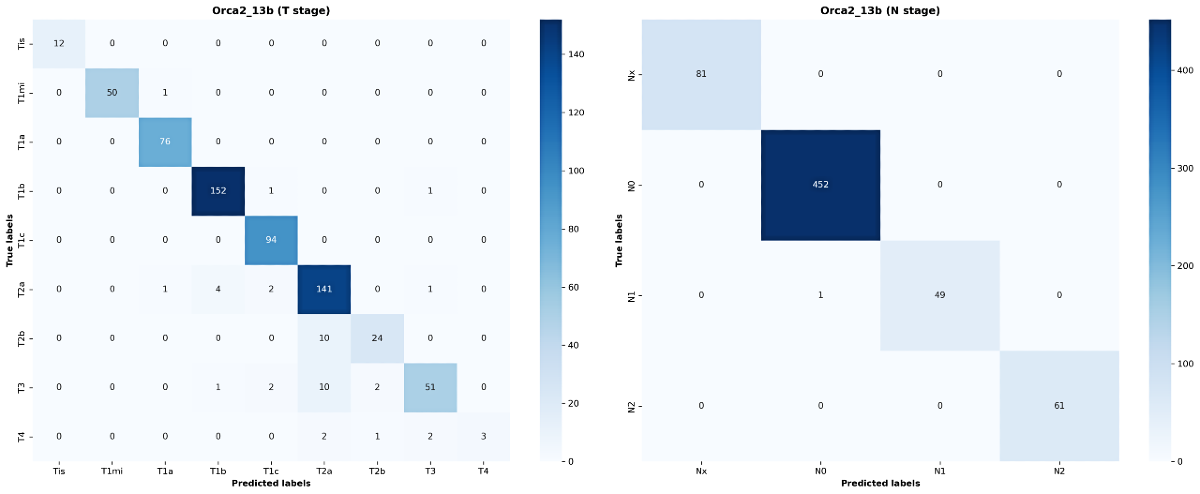
Confusion matrices for Orca2_13b model. (A) Confusion matrix for T stage results (Orca2_13b). (B) Confusion matrix for N stage results (Orca2_13b).

Multimedia Appendix 3 shows the performance of each model for TN classification, along with the count for each class. For the T classification, the Orca2_13b model achieved high *F*_1_-scores of 1.000, 0.990, 0.987, 0.977, 0.974, and 0.903 for Tis, T1mi, T1a, T1b, T1c, and T2a, respectively. The *F*_1_-scores for T2b and T3 were 0.786 and 0.842, respectively, whereas that for T4 was 0.545, indicating the highest precision but markedly low reproducibility. Figure 3 shows the confusion matrix of the Orca2_13b model. Confusion matrices for the other models are provided in Multimedia Appendix 4.

**Table 2:** EMR^a^ results for each model in predicting T and N classifications independently, as well as combined TN classification.

Model	T category EMR	N category EMR	TN categories EMR
Orca2_13b	0.936	0.998	0.934
Orca2_7b	0.917	0.996	0.914
Mistral_7b	0.577	0.788	0.572
Llama2_7b	0.872	0.973	0.864
Llama2_13b	0.765	0.869	0.762
Llama3_8b	0.538	0.883	0.489

^a^EMR: exact match ratio.

The Orca2_7b model achieved *F*_1_-scores of 0.781, 0.698, and 0.714 for T2b, T3, and T4, respectively, indicating low reproducibility for T4. The Llama2_13b model showed a higher *F*_1_-score for T4 than the other models; however, considerably low *F*_1_-scores were observed for Tis and T1mi.

The Orca2_13b model achieved *F*_1_-scores of 1.000, 0.998, 0.989, and 1.000 for N classification. This was followed by the Orca2_7b model, which yielded *F*_1_-scores of 0.987, 1.000, 0.990, and 0.991. This model performed slightly better than the Orca2_13b model for N0 and N1. In contrast, the Llama3_8b model performed worse than the other models for N1 and N2, with *F*_1_-scores of 0.531 and 0.584, respectively.

### OOS Analysis

Table 3 presents the OOS results for TN classification. Both the Orca2_13b and Orca2_7b models achieved OOS ratios of 0% for the T and N classifications. In contrast, the Mistral_7b, Llama2_7b, Llama2_13b, and Llama3_8b models had OOS ratios of 19.88%, 2.17%, 12.58%, and 12.73% for the T classification, as well as 20.34%, 1.40%, 12.42%, and 2.64% for the N classification, respectively.

**Table 3:** OOS^a^ ratios for pathologic TN classification for each model, showing the number and percentage of cases classified as OOS for T and N categories.

Model	T category			N category	
	OOS cases, n	OOS ratio (%)	OOS cases, n		OOS ratio (%)
Orca2_13b	0	0.00	0		0.00
Orca2_7b	0	0.00	0		0.00
Mistral_7b	128	19.88	131		20.34
Llama2_7b	14	2.17	9		1.40
Llama2_13b	81	12.58	80		12.42
Llama3_8b	82	12.73	17		2.64

^a^OOS: out-of-scope.

### Performance on Rationale Generation

Table 4 shows the performance evaluation of the GLMs, considering both the pathologic TN classification and rationale parts generated by the model. This evaluation was used as a measure of the ability of the model to accurately and logically present TN classification predictions and their rationale. The rationale part was measured when a sentence was completely consistent with the reference answer and when it had the same meaning in context. The Orca2_13b model achieved the best performance, with an EMR of 0.863 and an SMR of 0.864. The Orca2_7b model also performed well, achieving EMR and SMR of 0.84. These results highlight the consistent and accurate predictions of TN classification and the presentation of evidence by the Orca2 model. The Llama2_7b model achieved an EMR of 0.636 and an SMR of 0.771, whereas the Llama2_13b model achieved an EMR of 0.681 and an SMR of 0.690. In contrast, the Mistral_7b model had an EMR of 0.262 and an SMR of 0.377. The Llama3_8b model exhibited the lowest performance, with an EMR of 0.187 and an SMR of 0.456.

**Table 4:** Performance of each model in pathologic TN classification prediction and rationale generation, showing EMR^a^ and SMR^b^.

Model	EMR	SMR
Orca2_13b	0.863	0.864
Orca2_7b	0.854	0.854
Mistral_7b	0.262	0.377
Llama2_7b	0.636	0.771
Llama2_13b	0.681	0.690
Llama3_8b	0.187	0.456

^a^EMR: exact match ratio.

^b^SMR: semantic match ratio.

## Discussion

### Principal Findings

This study proposes a method that leverages lightweight GLMs to automatically infer pathological TN classification from lung cancer surgical pathology reports and provide corresponding rationales for these classifications. The high performance of the Orca2 model demonstrates its potential to reduce the human effort and errors associated with complex cancer staging tasks. Additionally, by generating rationales, the model provides interpretable results that enhance human trust. Furthermore, this study confirms the feasibility of fine-tuning these models to update and apply the latest cancer staging guidelines in environments with limited computing resources, such as medical institutions, enabling accurate staging and effective cancer data curation.

In this study, CoT fine-tuning played a crucial role in enabling the GLMs to clearly generate the rationale for TN classification. CoT fine-tuning allowed the model to generate intermediate reasoning steps in a step-by-step manner, reflecting logical thought processes for complex clinical texts. This enabled the model to provide rationale based not only on simple classification results but also on considering various clinical factors such as tumor size and lymph node status. In contrast to traditional rule-based NLP approaches, which rely on predefined rules for information extraction and fail to explicitly present complex reasoning steps, CoT’s automated processing of these steps allows for a clear presentation of the rationale. This step-by-step methodology enhances the interpretability of predictions, making the advantages of CoT fine-tuning particularly evident in complex clinical decision-making tasks, such as TN classification.

Determining T and N classifications based on lung cancer surgical pathology report information can prove difficult in the prediction of TN classification. Surgical pathology reports contain disparate information that must be integrated and inferred to derive a TN classification. The T classification must be derived based on overall judgments of the maximum tumor size, multiplicity (expressed in terms of satellites and separate nodules), location, and scope of invasion. The N classification is based on the precise position of lymph node metastases (eg, mediastinal or hilar) and the presence or absence of contralateral lymph node metastases. According to the recently suggested TNM classification, 9th edition [33], further subclassification as N2a or N2b can be performed depending on whether N2 lymph node metastases are present in a single station or multiple stations. This indicates the importance of the precise location of lymph node stations showing tumor involvement.

All models poorly predicted T4, which is typically caused by invasion of adjacent organs such as the esophagus, heart, or aorta. Surgery may not be feasible in these cases, resulting in an absence of surgical pathology reports. Additionally, cases classified as T4 may have ipsilateral lung metastasis; however, information regarding this metastasis is often omitted from surgical pathology reports in patients who only undergo partial surgery for diagnostic purposes. Therefore, we concluded that the challenges in predicting T4 were due to T4 classification-related factors that were not described in the surgical pathology reports. Furthermore, the NCCN guidelines recommend definite concurrent chemoradiation as an initial treatment for T4 cases, except for clinically resectable tumors. As this study only included patients who had undergone surgery, the ratio of T4 patients was relatively low. T4 cases accounted for only 2.51% of the total dataset in this study, which was insufficient compared to the other stages. Consequently, it was challenging to make generalizations during model training.

For T2b, which exhibited somewhat lower performance, all predictions except the correct ones were classified as T2a instead of T2b. In all 10 failed predictions (Figure 3), the models extracted the phrase “invasion to visceral pleura,” which is evidence that is common to both T2a and T2b. However, the maximum tumor size that differentiates T2a from T2b often caused ambiguity in this study. T classification was performed through consultation with a pathology expert based on the size of the tumor bed and that of the invasive component for mucinous- and nonmucinous-type lung cancer, respectively. However, the actual dataset revealed that T classification was often derived based on the invasive component size for both lung cancer types, which caused several nonmatches. Thus, confusion regarding the use of the maximum tumor size criterion may have caused problems with generalization when differentiating between T2a and T2b. Model performance was better for T2a than for T2b because T2a accounted for 23.69% of the total dataset, which is approximately four times higher than that of T2b (5.03%). Therefore, T2a appeared more generalized during training. N1 and N2 are at the same lung cancer stage for either T2a or T2b of IIb (T2aN1, T2bN1) or IIIA (T2aN2, T2bN2). Therefore, they have no clinical effect on the treatment plan or prognosis. For N0, the stage differs between T2a and T2b (IB and IIA); however, the recommended primary treatment in the NCCN guidelines is surgery for both. Therefore, the poor performance in differentiating T2b has relatively little clinical impact. Although the poor performance in distinguishing between T2a and T2b has a low clinical impact, the differentiation of T categories remains clinically significant. The study [34], following the *AJCC Cancer Staging Manual 8th edition* criteria, confirmed that each T category—based on tumor size increments and specific characteristics—significantly impacts survival outcomes. For instance, tumors over 5 cm but not exceeding 7 cm are associated with a T3 prognosis, while those over 7 cm align with T4. Furthermore, the findings demonstrated that even small increments in tumor size (from 1 to 5 cm) are associated with distinctly different prognoses, emphasizing the clinical importance of precise T classification adjustments. Examples of correctly and incorrectly classified cases from the Orca2_13 model, including cases such as T4 and T2b, are provided in Multimedia Appendix 5.

Most models demonstrated excellent performance during precise N classification. The Orca2_13b model demonstrated an outstanding performance, accurately predicting all but one case. Moreover, the data were evenly distributed among the classes for N classification, indicating that various classes were well-represented during training.

The OOS predictions observed in this study are an important factor for evaluating the models. We found that the Orca2 model consistently generated predictions within a defined range.

The result of this study indicates that GLMs, such as Orca2, can enhance the accuracy of staging and improve diagnostic consistency by automatically providing TN classification information in clinical and research settings. Furthermore, they have the potential to reduce human effort, time, and errors associated with complex cancer staging analyses. This study’s approach also has the potential to impact training for health care professionals and standardize staging practices across hospitals. By automatically providing accurate TN classifications along with interpretable rationales based on specific AJCC criteria, these models can serve as an educational tool for both specialists and nonspecialist medical staff, helping them understand and apply the latest guidelines. Furthermore, the consistency achieved through GLMs can reduce discrepancies across institutions, thereby improving the overall reliability and standardization of cancer staging data.

### Limitations and Future Works

This study was based on lung cancer surgical pathology report data collected from a single tertiary general hospital. Due to the limitations of using a single data source, the generalizability of the results may be limited. Data from a single hospital may not fully capture the diversity of patient characteristics, cancer subtypes, or variations in report details and formats. Future studies should incorporate multicenter data to enhance the performance of the proposed method and assess its flexibility and scalability by applying it to pathology reports in varying formats from different hospitals. Such efforts will improve the model’s robustness and evaluate its applicability across diverse clinical environments.

Especially, the limited data for T4 and T2b classifications posed constraints on model training in this study. T4 is diagnosed based on specific factors such as invasion of surrounding structures or ipsilateral lung metastasis, but such information is often not included in surgical pathology reports, and the number of T4 patients undergoing surgery is also low, resulting in insufficient data. Similarly, T2b classification varies depending on the mucinous versus nonmucinous subtype of lung cancer, but in the training data, mixed criteria for maximum tumor size made it difficult to accurately distinguish between T2a and T2b. Future studies could consider improving the model’s generalization performance by acquiring additional patient cases for T4 and T2b classifications from diverse medical institutions, using data augmentation techniques, or generating synthetic datasets to address these limitations.

Additionally, in environments with limited computing resources, model size is a critical factor influencing performance. In this study, we used relatively small GLMs to prioritize efficiency. However, if the computational demands of larger models can be reduced through advanced techniques such as knowledge distillation or quantization, high predictive performance could still be achieved in resource-constrained clinical settings. These approaches offer promising strategies for optimizing models to balance performance and efficiency effectively.

The TN classification approach proposed in this study is worth exploring for application to other cancers, such as breast cancer or colorectal cancer, where pathological staging is crucial. This could further demonstrate the potential applicability and versatility of the model across a broader range of contexts.

### Conclusions

In this study, we propose a new methodology using GLMs to infer pathologic TN classification and generate rationales based on the *AJCC Cancer Staging Manual 8th edition* criteria. Orca2, enhanced with CoT fine-tuning, achieved the highest accuracy in TN classification and rationale generation, demonstrating its potential to streamline staging processes in lung cancer pathology. Automatic pathologic TN classification has the potential to reduce cancer staging time while enhancing accuracy and consistency in both clinical and research settings. Despite its relatively small size as a 7-billion-parameter model, this study highlights the efficiency and effectiveness of lightweight language models for analyzing health care data in medical settings with limited computing resources.

The GLM-based approach proposed in this study has the capability to advance artificial intelligence–assisted clinical diagnosis and contribute to the standardization of complex cancer staging tasks, facilitating the creation of consistent oncological data across diverse medical institutions. To further assess the model’s flexibility and scalability, future research should focus on validating its performance using multicenter datasets, thereby exploring its applicability across various clinical settings. Additionally, this would allow for the exploration of the proposed method’s potential for use in other cancer types, beyond lung cancer pathology.

## Appendix

Multimedia Appendix 1 TN stage classification and rationale.

Multimedia Appendix 2 Summary of experimental setup for large language model training.

Multimedia Appendix 3 Pathologic TN classification performance of each model, with precision, recall, and F1-scores for individual T and N categories.

Multimedia Appendix 4 Confusion matrix results for the TN stage classification prediction of each model.

Multimedia Appendix 5 Examples of correctly and incorrectly classified cases from Oracl2_13 model.

## Abbreviations

AJCC: American Joint Committee on Cancer
BERT: Bidirectional Encoder Representations from Transformers
CoT: chain-of-thought
EHR: electronic health record
EMR: exact match ratio
GLM: generative language model
LLM: large language model
NCCN: National Comprehensive Cancer Network
NER: named entity recognition
NLP: natural language processing
OOS: out-of-scope
SMR: semantic match ratio
SNUBH: Seoul National University Bundang Hospital
