# Supplementary material for: Automated Pathologic TN Classification Prediction and Rationale Generation From Lung Cancer Surgical Pathology Reports Using a Large Language Model Fine-Tuned With Chain-of-Thought: Algorithm Development and Validation Study
Source: JMIR Med Inform. 2024 Dec 20;12:e67056. doi: 10.2196/67056 (PMC11699504; doi:10.2196/67056)
Supplement: Multimedia Appendix 1 [file medinform_v12i1e67056_app1.docx]

**TN stage classification and rationale**

| Stage Category | Rationale |
| --- | --- |
| Tis | - It is one of three types: carcinoma in situ, squamous cell carcinoma in situ (SCIS), or adenocarcinoma in situ (AIS). |
| T1mi | - Minimally invasive adenocarcinoma. |
| T1a | - Max tumor size is less than or equal to 1cm. |
| T1b | - Max tumor size is less than or equal to 2 cm and larger than 1 cm. |
| T1c | - Max tumor size is less than or equal to 3 cm and larger than 2 cm. |
| T2a | - Max tumor size is greater than 3cm and less than or equal to 4cm.  - Invasion to visceral pleural.  - Involves the main bronchus regardless of distance to the carina, but without involvement of the carina.  - Associated with atelectasis or obstructive pneumonitis that extends to the hilar region, involving part or all of the lung. |
| T2b | - Max tumor size is greater than 4cm and less than or equal to 5cm.  - Invasion to visceral pleural.  - Involves the main bronchus regardless of distance to the carina, but without involvement of the carina.  - Associated with atelectasis or obstructive pneumonitis that extends to the hilar region, involving part or all of the lung. |
| T3 | - Max tumor size is greater than 5cm and less than or equal to 7cm.  - Invasion to chest wall.  - Invasion to parietal pleura.  - Invasion to pericardium.  - Invasion to phrenic nerve.  - Two or more separate tumor nodules in the same lobe as the primary tumor.  - Lung to lung metastasis.  - Intrapulmonary metastasis.  - Same lobe as primary satellite.  - Same lobe as primary separate. |
| T4 | - Max tumor size is greater than 7cm.  - Invasion to mediastinum.  - Invasion to diaphragm.  - Invasion to heart.  - Invasion to great vessels.  - Invasion to recurrent laryngeal nerve.  - Invasion to trachea.  - Invasion to esophagus.  - Invasion to vertebral body(spines).  - Metastatic right upper lobe.  - Metastatic right middle lobe.  - Metastatic right lower lobe.  - Metastatic left upper lobe.  - Metastatic left lower lobe.  - Invasion to aorta.  - Invasion to SVC(Superior vena cava).  - Invasion to IVC(Inferior vena cava).  - Invasion to pulmonary artery.  - Invasion to pulmonary vein.  - Invasion to carina. |
| Nx | - Regional lymph node metastasis information is missing or not submitted. |
| N0 | - No regional lymph node metastasis. |
| N1 | - There was metastasis to the intrapulmonary lymph nodes of the right lung.  - There was metastasis to the intrapulmonary lymph nodes of the left lung.  - There was metastasis to the hilar lymph nodes of the right lung.  - There was metastasis to the hilar lymph nodes of the left lung. |
| N2 | - There was metastasis to the mediastinal lymph nodes of the right lung.  - There was metastasis to the mediastinal lymph nodes of the left lung. |
