# Supplementary material for: Automated Pathologic TN Classification Prediction and Rationale Generation From Lung Cancer Surgical Pathology Reports Using a Large Language Model Fine-Tuned With Chain-of-Thought: Algorithm Development and Validation Study
Source: JMIR Med Inform. 2024 Dec 20;12:e67056. doi: 10.2196/67056 (PMC11699504; doi:10.2196/67056)
Supplement: Multimedia Appendix 2 [file medinform_v12i1e67056_app2.docx]

**Summary of experimental setup for LLM training. The hyperparameters used in model training and evaluation are shown**

| Type | Parameter | Value |
| --- | --- | --- |
| LLM Training Parameters | Training Steps | 6000 |
|  | Batch Size per GPU | 2 |
|  | Gradient Accumulation Steps | 4 |
|  | Learning Rate | $1.5 \times{10}^{-5}$ |
|  | Optimizer | Paged AdamW 32bit |
| LoRA Parameters | LoRA Gamma | 32 |
|  | LoRA Alpha | 32 |
|  | LoRA Dropout | 0.05 |
| LLM Generation Parameters | Top-k | 1 |
|  | Number of Beams | 1 |
