# Supplementary material for: Automated Pathologic TN Classification Prediction and Rationale Generation From Lung Cancer Surgical Pathology Reports Using a Large Language Model Fine-Tuned With Chain-of-Thought: Algorithm Development and Validation Study
Source: JMIR Med Inform. 2024 Dec 20;12:e67056. doi: 10.2196/67056 (PMC11699504; doi:10.2196/67056)
Supplement: Multimedia Appendix 3 [file medinform_v12i1e67056_app3.docx]

Pathologic TN classification performance of each model, with precision, recall, and *F*_1_-scores for individual T and N categories.

| Categories | Orca2_13b^a^ | | | Orca2_7b^b^ | | | Mistral_7b^c^ | | | Llama2_7b^d^ | | | Llama2_13b^e^ | | | Llama3_8b^f^ | | | |
| --- | --- | --- | --- | --- | --- | --- | --- | --- | --- | --- | --- | --- | --- | --- | --- | --- | --- | --- | --- |
|  | Precision | Recall | F1 | Precision | Recall | F1 | Precision | Recall | F1 | Precision | Recall | F1 | Precision | Recall | F1 | Precision | Recall | F1 |  |
| Tis (12) | 1.000 | 1.000 | 1.000 | 1.000 | 1.000 | 1.000 | 1.000 | 0.083 | 0.153 | 1.000 | 0.083 | 0.153 | 0.000 | 0.000 | 0.000 | 0.600 | 0.500 | 0.545 |  |
| T1mi (51) | 1.000 | 0.980 | 0.990 | 1.000 | 0.980 | 0.990 | 0.875 | 0.137 | 0.237 | 1.000 | 0.745 | 0.853 | 0.500 | 0.019 | 0.037 | 0.875 | 0.137 | 0.237 |  |
| T1a (76) | 0.974 | 1.000 | 0.987 | 0.974 | 0.986 | 0.980 | 0.240 | 0.263 | 0.251 | 0.800 | 1.000 | 0.888 | 0.648 | 0.631 | 0.640 | 0.370 | 0.973 | 0.536 |  |
| T1b (154) | 0.968 | 0.987 | 0.977 | 0.968 | 0.993 | 0.980 | 0.806 | 0.623 | 0.703 | 0.954 | 0.954 | 0.954 | 0.933 | 0.915 | 0.924 | 0.975 | 0.512 | 0.672 |  |
| T1c (94) | 0.949 | 1.000 | 0.974 | 0.946 | 0.936 | 0.941 | 0.957 | 0.712 | 0.817 | 0.906 | 0.925 | 0.915 | 0.854 | 0.872 | 0.863 | 0.688 | 0.563 | 0.619 |  |
| T2a (149) | 0.865 | 0.946 | 0.903 | 0.820 | 0.979 | 0.892 | 0.792 | 0.872 | 0.830 | 0.875 | 0.946 | 0.909 | 0.938 | 0.919 | 0.928 | 0.750 | 0.503 | 0.602 |  |
| T2b (34) | 0.888 | 0.705 | 0.786 | 0.833 | 0.735 | 0.781 | 0.629 | 0.500 | 0.557 | 0.965 | 0.823 | 0.888 | 0.848 | 0.823 | 0.835 | 0.447 | 0.500 | 0.472 |  |
| T3 (66) | 0.927 | 0.772 | 0.842 | 0.925 | 0.560 | 0.698 | 0.833 | 0.454 | 0.588 | 0.788 | 0.621 | 0.694 | 0.943 | 0.757 | 0.840 | 0.769 | 0.454 | 0.571 |  |
| T4 (8) | 1.000 | 0.375 | 0.545 | 0.833 | 0.625 | 0.714 | 0.500 | 0.500 | 0.500 | 0.750 | 0.375 | 0.500 | 0.750 | 0.750 | 0.750 | 0.666 | 0.750 | 0.705 |  |
| Nx (81) | 1.000 | 1.000 | 1.000 | 1.000 | 0.975 | 0.987 | 1.000 | 0.419 | 0.591 | 1.000 | 0.888 | 0.941 | 1.000 | 0.135 | 0.239 | 0.983 | 0.753 | 0.853 |  |
| N0 (452) | 0.997 | 1.000 | 0.998 | 1.000 | 1.000 | 1.000 | 0.992 | 0.851 | 0.916 | 0.986 | 0.993 | 0.990 | 0.995 | 0.973 | 0.984 | 0.959 | 0.995 | 0.977 |  |
| N1 (50) | 1.000 | 0.980 | 0.989 | 0.980 | 1.000 | 0.990 | 0.947 | 0.720 | 0.818 | 1.000 | 0.960 | 0.979 | 1.000 | 0.980 | 0.989 | 0.568 | 0.500 | 0.531 |  |
| N2 (61) | 1.000 | 1.000 | 1.000 | 0.983 | 1.000 | 0.991 | 1.000 | 0.868 | 0.929 | 0.966 | 0.950 | 0.958 | 0.967 | 0.983 | 0.975 | 0.634 | 0.540 | 0.584 |  |

^a^T category macro *F*_1_-score: 0.889; N category macro *F*_1_-score: 0.997.

^b^T category macro *F*_1_-score: 0.886; N category macro *F*_1_-score: 0.992.

^c^T category macro *F*_1_-score: 0.515; N category macro *F*_1_-score: 0.888.

^d^T category macro *F*_1_-score: 0.750; N category macro *F*_1_-score: 0.967.

^e^T category macro *F*_1_-score: 0.646; N category macro *F*_1_-score: 0.797.

^f^T category macro *F*_1_-score: 0.551; N category macro *F*_1_-score: 0.736.
