# Supplementary figures and images for: Automated Pathologic TN Classification Prediction and Rationale Generation From Lung Cancer Surgical Pathology Reports Using a Large Language Model Fine-Tuned With Chain-of-Thought: Algorithm Development and Validation Study
Source: JMIR Med Inform. 2024 Dec 20;12:e67056. doi: 10.2196/67056 (PMC11699504; doi:10.2196/67056)

**
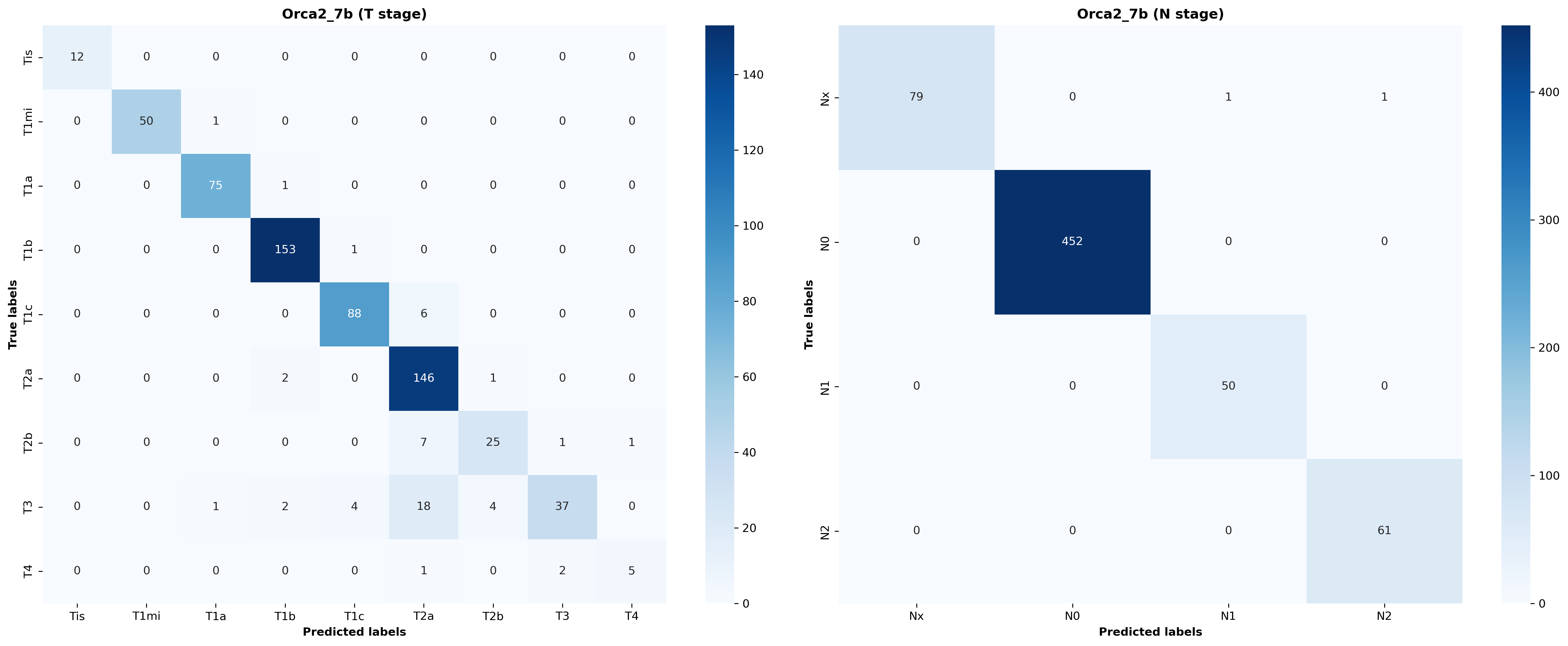
**


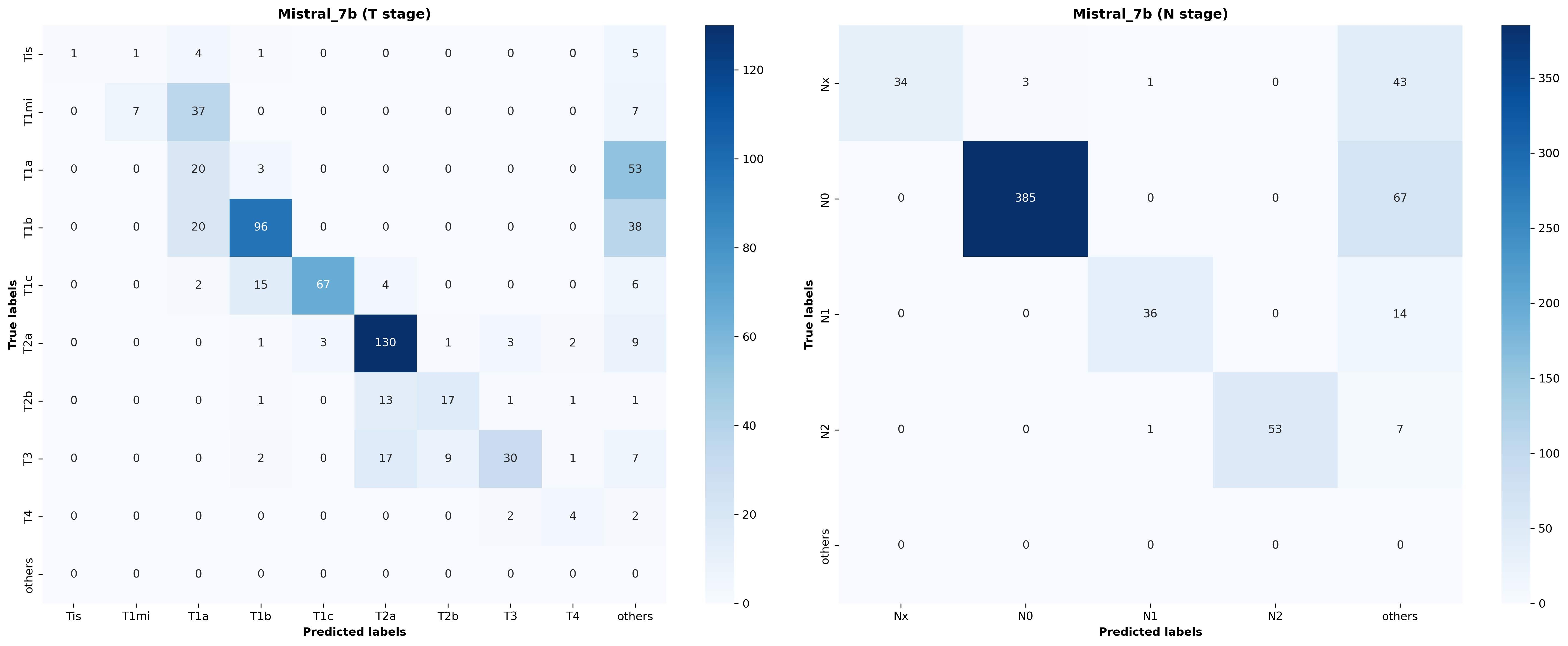


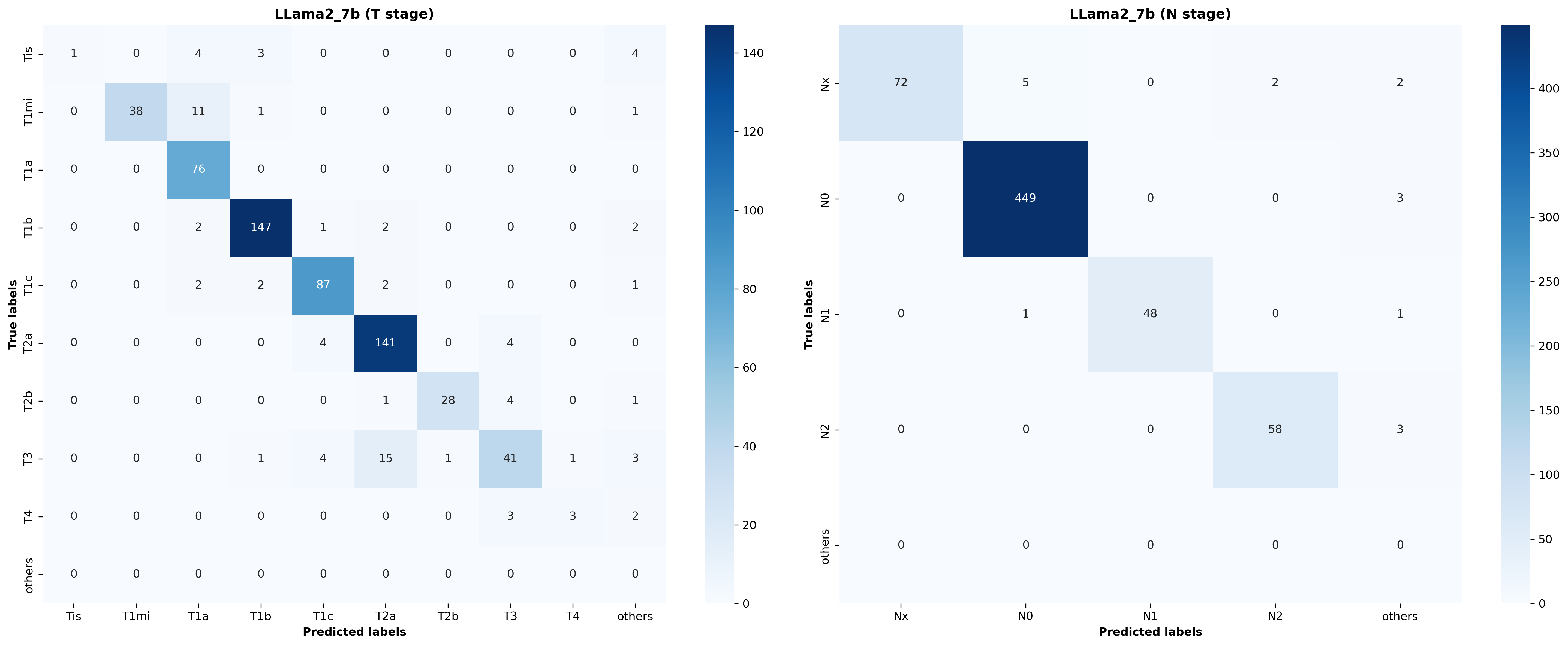


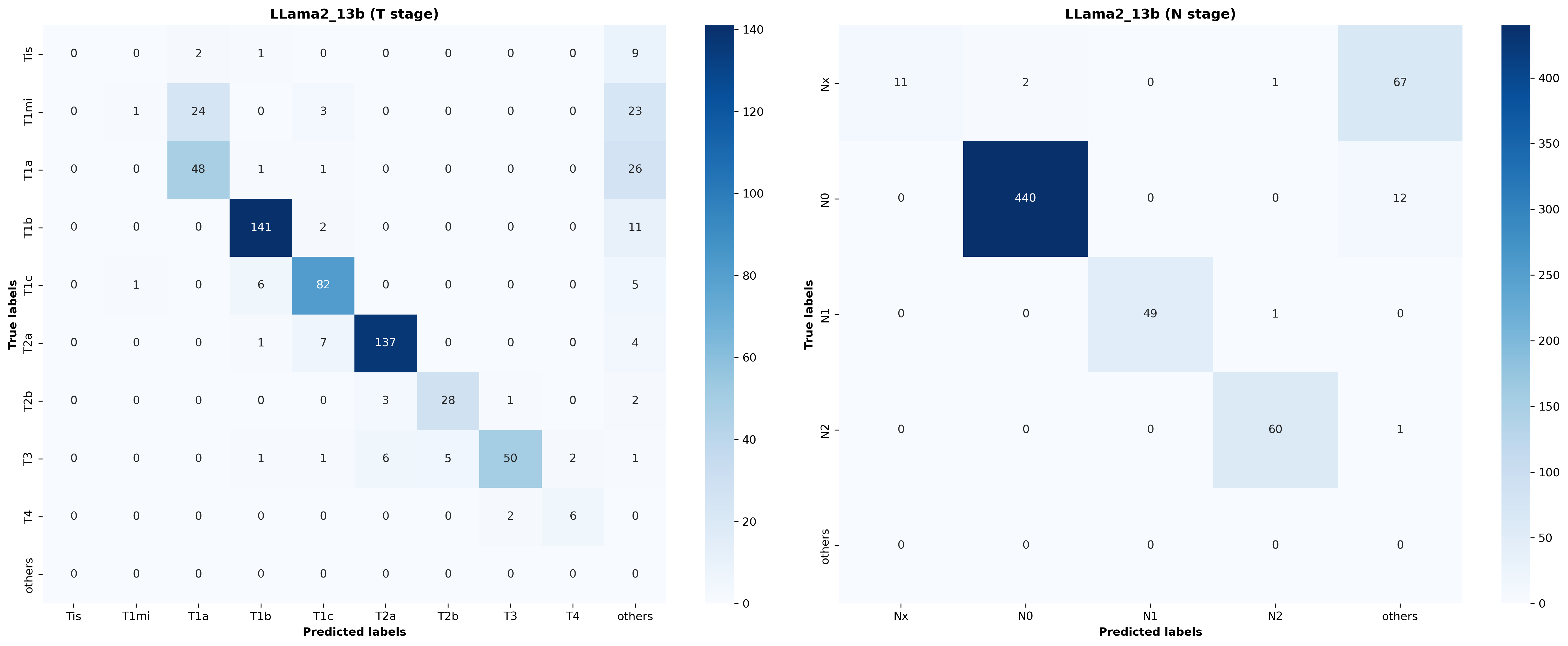


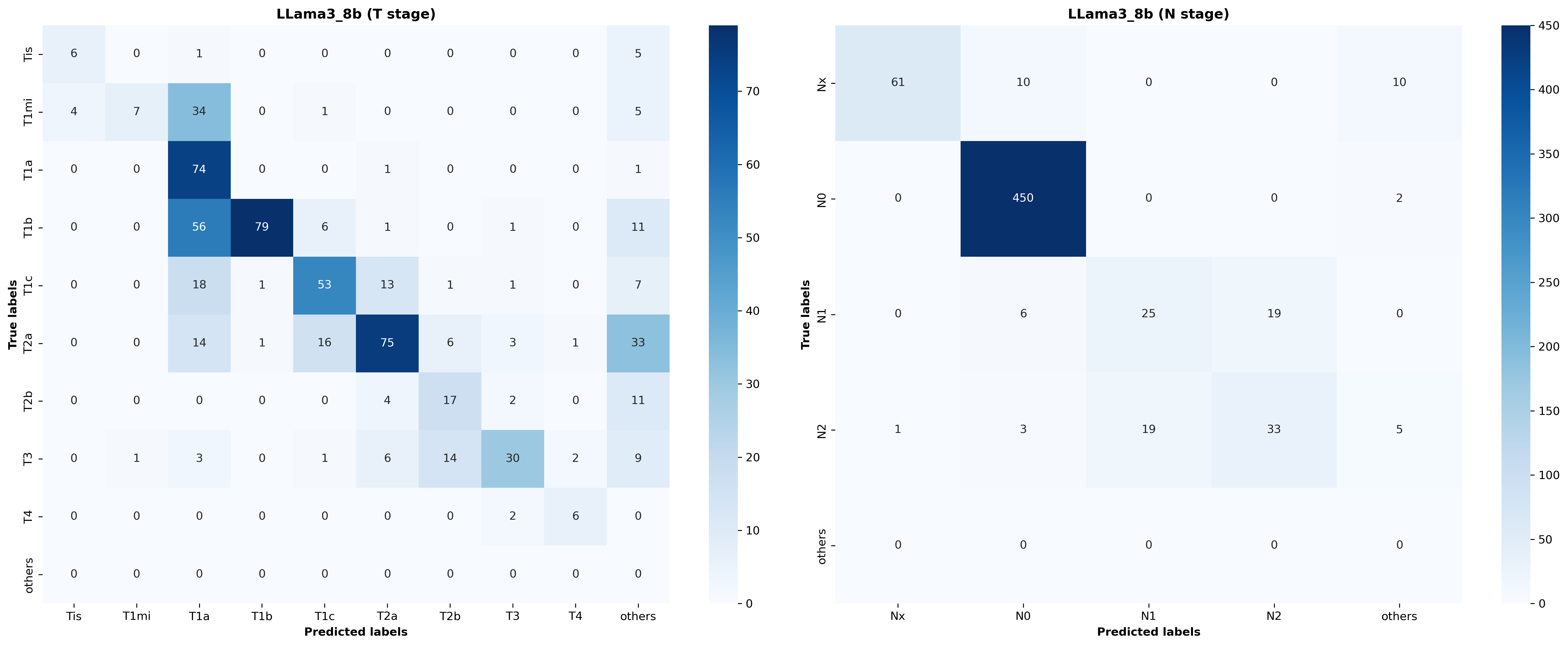

Supplement: Multimedia Appendix 4 [file medinform_v12i1e67056_app4.docx]
