# Supplementary material for: Automated Pathologic TN Classification Prediction and Rationale Generation From Lung Cancer Surgical Pathology Reports Using a Large Language Model Fine-Tuned With Chain-of-Thought: Algorithm Development and Validation Study
Source: JMIR Med Inform. 2024 Dec 20;12:e67056. doi: 10.2196/67056 (PMC11699504; doi:10.2196/67056)
Supplement: Multimedia Appendix 5 [file medinform_v12i1e67056_app5.pdf]

Examples of correctly and incorrectly classified cases from Oracl2_13 model

Model Name	Case Type	Case Description	Analysis
Orca2_13b	Correct Classification	**<Test Case Report #1>**<br>[A] Lung, left lower lobe, lobectomy :<br>ADENOCARCINOMA, micropapillary predominant [by 2015 WHO classification]<br>- other subtypes : acinar (10%), solid (20%)<br>1) Preoperative / Previous treatment: not done<br>2) Histologic grade: 3 (micropapillary or solid)<br>3) Micropapillary pattern: PRESENT<br>4) Nuclear grade: 3<br>5) Size of tumor: 1.5 x 1.2 x 1.0cm<br>6) Extent of invasion<br>a) Invasion to visceral pleura: PRESENT (P1)<br>b) Invasion to parietal pleura: not submitted<br>c) Invasion to diaphragm: not submitted<br>7) Main bronchus: not involved<br>8) Vascular invasion: PRESENT<br>9) Lymphatic invasion: PRESENT<br>10) Perineural invasion: not identified<br>11) Inflammatory reaction: lymphoplasmacytic<br>12) Aerogenous spread: PRESENT (grade 2)<br>13) Necrosis: PRESENT (10%)<br>14) Resection margin: free from carcinoma (safety margin: 3.2cm)<br>15) Lymph node: submitted<br>a) metastasis in 7 out of 25 regional lymph nodes<br>(peribronchial lymph node: 0/3, LN#5,6: 0/3, LN#4: 0/1, LN#7: 0/4, LN#9: 0/3, LN#10: 0/2, LN#11: 7/9)<br>b) extranodal extension:absent<br>17) Related slide: absent<br>18) Associated findings: absent<br>19) Other findings in background lung parenchyme: absent<br><br>{<br>"Reasoning1" : "Invasion to visceral pleural.",<br>"Reasoning2" : "There was metastasis to the hilar lymph nodes of the left lung.",<br>"Answer" : "pT2aN1"<br>}	Accurately identified visceral pleural invasion, upstaging the tumor to pT2a, and recognized lymph node metastasis to LN#11, correctly classifying as pN1.
Orca2_13b	Incorrect Classification	**<Test Case Report #2>**<br>[A] Lung, right lower lobe, lobectomy :<br>INVASIVE MUCINOUS ADENOCARCINOMA, acinar predominant [by 2015 WHO classification]<br>- other subtypes : micropapillary (20%), solid (15%)<br>1) Preoperative / Previous treatment: not done<br>2) Histologic grade: 2 (acinar/papillary etc)<br>3) Micropapillary pattern: PRESENT<br>4) Nuclear grade: 3<br>5) Size of tumor: 7.3 x 6.3 x 3.6cm (Tm1), 2.2 x 1.8 x 1.0cm (Tm2)<br>a) Invasive component only: 7.3 x 6.3 x 3.6cm (Tm1), 2.2 x 1.8 x 1.0cm (Tm2)<br>b) Including CIS (=former BAC) component: 7.3 x 6.3 x 3.6cm (Tm1), 2.2 x 1.8 x 1.0cm (Tm2)<br>6) Extent of invasion<br>a) Invasion to visceral pleura: absent (P0)<br>b) Invasion to parietal pleura: not submitted<br>c) Invasion to diaphragm: not submitted<br>7) Main bronchus: not submitted<br>8) Vascular invasion: not identified<br>9) Lymphatic invasion: not identified<br>10) Perineural invasion: not identified<br>11) Inflammatory reaction: lymphoplasmacytic, neutrophilic<br>12) Aerogenous spread: PRESENT (grade 2)<br>13) Necrosis: PRESENT (5%)<br>14) Resection margin: free from carcinoma (safety margin: 1.5cm)<br>15) Lymph node: submitted<br>a) no metastasis in 5 regional lymph nodes<br>(peribronchial lymph node: 0/3, LN #7: 0/1, LN #11M: 0/1)<br>17) Related slide: S20-34249: Non-small cell carcinoma<br>18) Associated findings: satellite nodule<br>19) Other findings in background lung parenchyme:<br>Subpleural and interstitial fibrosis<br>with 1) honeycombing change<br>2) temporal and spatial heterogeneity	Misclassified the tumor as **pT3N0** based on the presence of a satellite nodule, but overlooked that the tumor size (>7 cm) upstages it to **pT4N0** according to AJCC 8th edition criteria.

		consistent with Usual interstitial pneumonia (UIP) pattern<br>{<br>"Reasoning1" : "Same lobe as primary satellite.",<br>"Reasoning2" : "No regional lymph node metastasis.",<br>"Answer" : "pT3N0"<br>}	
Orca2_13b	Incorrect Classification	**<Test Case Report #3>**<br>[A] Lung, right upper lobe, lobectomy :<br>Mixed invasive mucinous adenocarcinoma and non-mucinous adenocarcinoma, acinar predominant [by 2021 WHO classification]<br>- other subtypes : papillary(10%)<br>1) Preoperative / Previous treatment: not done<br>2) Histologic grade: moderately differentiated (acinar, papillary)<br>3) Micropapillary pattern: absent<br>4) Nuclear grade: 3<br>5) Size of tumor:<br>a) Invasive component only: 4.3 x 2.8 x 2.0cm<br>b) Including AIS (=former BAC) component: 4.3 x 2.8 x 2.0cm<br>6) Extent of invasion<br>a) Invasion to visceral pleura: PRESENT (P2)<br>b) Invasion to parietal pleura: not submitted<br>c) Invasion to diaphragm: not submitted<br>7) Main bronchus: not submitted<br>8) Vascular invasion: not identified<br>9) Lymphatic invasion: PRESENT<br>10) Perineural invasion: not identified<br>11) Inflammatory reaction: not identified<br>12) Aerogenous spread: not identified<br>13) Necrosis: PRESENT (15%)<br>14) Resection margin: free from carcinoma (safety margin: 4.0cm)<br>15) Lymph node: submitted<br>a) metastasis in 2 out of 18 regional lymph nodes<br>(peribronchial lymph node: 1/5, LN#R2,3,4: 1/6, LN#7: 0/7)<br>b) extranodal extension: absent<br>17) Related slide: absent<br>18) Associated findings: extracellular mucin<br>19) Other findings in background lung parenchyme: subpleural fibrosis and emphysematous change<br>{<br>"Reasoning1" : "Invasion to visceral pleural.",<br>"Reasoning2" : "There was metastasis to the mediastinal lymph nodes of the right lung.",<br>"Answer" : "pT2aN2"<br>}	Correctly identified visceral pleural invasion and N2 lymph node metastasis, but misclassified the tumor size (>4 cm ≤5 cm) as **pT2a** instead of **pT2b**, leading to an incorrect overall classification of **pT2aN2**.
